# Network pharmacology to explore the mechanism of scutellarin in the treatment of brain ischaemia and experimental verification of JAK2/STAT3 signalling pathway

**DOI:** 10.1038/s41598-023-33156-5

**Published:** 2023-05-09

**Authors:** Qiu-Ye Jia, Hao‑Lun Chen, Zhi Qi, Xiao‑Li‑Na Zhang, Li-Yang Zheng, Teng-Teng Liu, Yun Yuan, Li Yang, Chun‑Yun Wu

**Affiliations:** Department of Anatomy and Histology/Embryology, School of Basic Medical Sciences, Kunming Medical University, 1168 West Chunrong Road, Kunming, 650500 People’s Republic of China

**Keywords:** Computational biology and bioinformatics, Neuroscience

## Abstract

Scutellarin is used to treat brain ischaemia. However, its underlying mechanism of action remains unclear. This study aimed to elucidate the potential mechanism of action of scutellarin in brain ischaemia through network pharmacology and experimental verification. The JAK2/STAT3 signalling pathway was identified and experimentally verified. Expression of JAK2/STAT3 signalling related proteins in TNC-1 astrocytes with BV-2 microglia-conditioned medium (CM), CM + lipopolysaccharide (LPS) (CM + L), and CM pretreated with scutellarin + LPS (CM + SL) was analysed by Western Blot and immunofluorescence staining. Expression levels of JAK2, p-JAK2, STAT3, and p-STAT3 were evaluated in astrocytes pre-treated with AG490. Middle cerebral artery occlusion (MCAO) in rats was performed in different experimental groups to detect expression of the above biomarkers. Network pharmacology suggested that the JAK2/STAT3 signalling pathway is one of the mechanisms by which scutellarin mitigates cerebral ischaemic damage. In TNC-1 astrocytes*,* p-JAK2 and p-STAT3 expression were significantly up-regulated in the CM + L group. Scutellarin promoted the up-regulation of various markers and AG490 neutralised the effect of scutellarin. In vivo, up-regulation of p-JAK2 and p-STAT3 after ischaemia is known. These results are consistent with previous reports. Scutellarin further enhanced this upregulation at 1, 3, and 7 d after MCAO. Scutellarin exerts its therapeutic effects on cerebral ischaemia by activating the astrocyte JAK2/STAT3 signalling, which provides a firm experimental basis for its clinical application.

## Introduction

Stroke, which often leads to long-term disability and death, is a life-threatening condition, and approximately 85% of strokes are classified as ischaemic^1^. Ischaemic stroke is mainly caused by occlusion of the middle cerebral artery (MCA), which triggers a cascade of neuroinflammatory and immune responses^2^. Currently, the treatment options for patients with ischaemic stroke are limited. Recombinant tissue plasminogen activator (rt-PA) is an effective treatment for ischaemic stroke, although its neurotoxicity must be considered^3^. However, the applications of thrombolytic therapy are limited due to its short therapeutic time window and many contraindications^4^. Brain injury caused by ischaemic stroke results from a complex set of neuropathophysiological and neuropathological events, including oxidative stress, amyloid production, and tau protein dysfunction^5–7^. Excessive reactive oxygen species (ROS) produced by oxidative stress are the main cause of neuronal necrosis and apoptosis^8^, and scavenging excessive ROS effectively mitigates the progression of neuronal death and improves prognosis^9^.

Breviscapine is a crude extract of flavonoids of *Erigeron breviscapus (Vant.)* Hand.-Mazz*.* Scutellarin (4,5,6-trihydroxyflavone-7-glucuronide) is one of the representative flavone. Numerous studies have shown that many flavonoids have antioxidant activity and can improve patient survival^10–12^. Previous studies have shown that compared to edaravone, high-dose scutellarin (100 mg/kg) significantly reduces infarct volume after cerebral ischaemia in rats^13^. Fang et al.^14^ found that scutellarin significantly improved neurological function and reduced cerebral infarct volume in rats with experimentally induced cerebral ischaemia. Chen et al.^15^ demonstrated that scutellarin attenuates microglia-mediated neuroinflammation by downregulating inflammatory and pro-inflammatory mediators. However, the targets and mechanisms of action of scutellarin in the reduction of ischaemic brain injury remain unclear.

Recent studies have shown that microglia are the first cells to be activated following brain ischaemia. Activated microglia then swiftly migrate and accumulate in the infarct core and penumbral regions to phagocytose cellular debris. Activated microglia can also induce the activation of astrocytes, which are closely associated with microglia. The so-called “cross-talk” between activated microglia and astrocytes would consequently amplify the inflammatory cascade response^14^. Thus, the close temporal and spatial relationship between microglia and astrocytes at the lesion site of cerebral ischaemia may not be fortuitous. Indeed, cross-talk between microglia and astrocytes plays an important role in the pathophysiological process following brain ischaemia^16^.

The Janus kinase 2 (JAK2)/signal transducers and activators of transcription 3 (STAT3) signalling pathway is involved in ischaemia, metabolism, hypoxia, and oxidative stress in various tissues and organs. Many studies have reported that activation of the JAK2/STAT3 signalling pathway enhances cerebral angiogenesis and promotes neurological recovery after ischaemic stroke^17–19^. Hou et al.^20^ suggested that part of the mechanism underlying the neuroprotective effect of resveratrol on cerebral ischaemia/reperfusion injury is mediated by activation of the JAK2/STAT3 pathway. The JAK family and its associated receptors, the STAT family, are the three major components of the JAK2/STAT3 signalling pathway. Phosphorylated JAK2 (p-JAK2) specifically activates STAT3, and phosphorylated STAT3 (p-STAT3) forms a dimer that translocates from the cytoplasm to the nucleus, regulating the transcription of target genes and inducing corresponding gene expression^21^.

**Figure 1:**
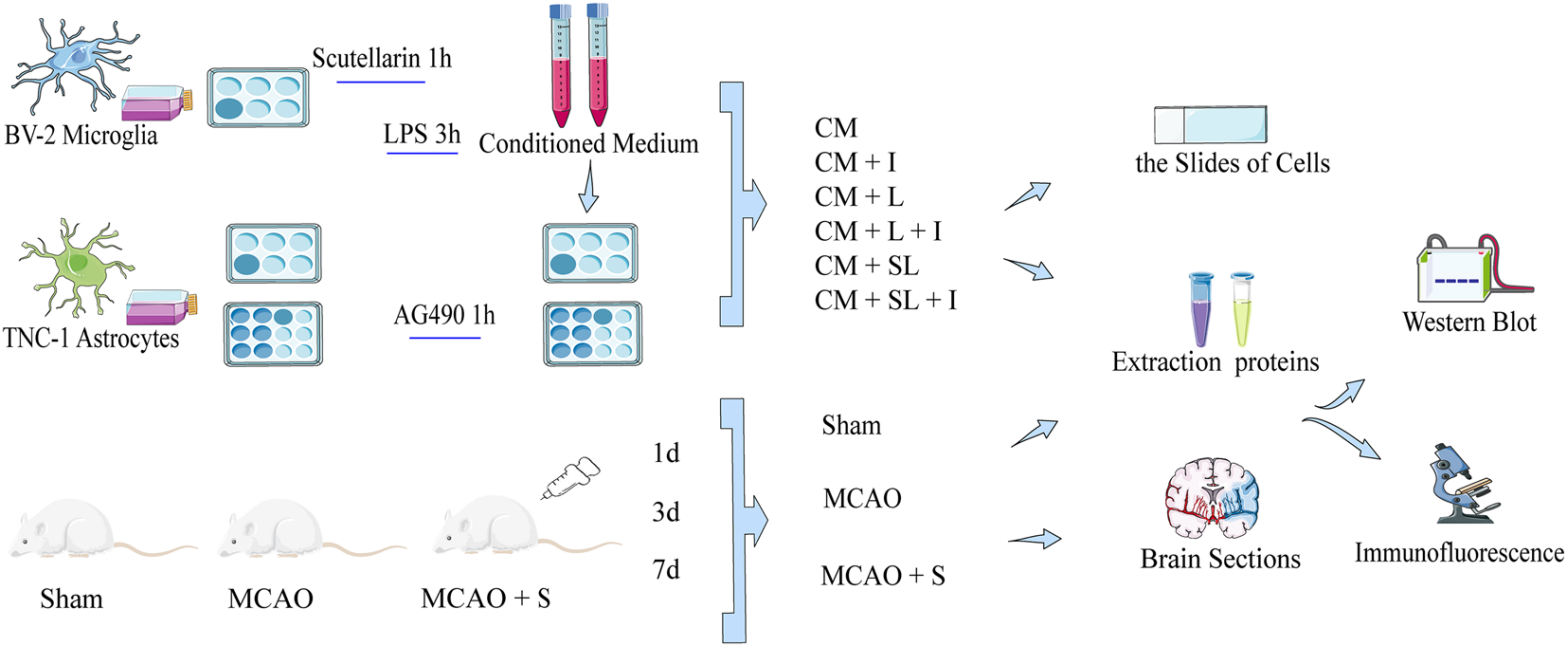
Experimental flow chart of this study.

To identify the new signalling pathways by which scutellarin attenuates brain ischaemic injury, we combined a network pharmacology approach and experimental validation. As intercellular cross-talk plays a crucial role in the treatment of cerebral ischaemia, we used BV-2 microglia-conditioned medium to incubate astrocyte in an in vitro experiment, which will help us better understand the process by which scutellarin treat brain ischaemia (Fig. 1).

## Results

### Integrated network of scutellarin and brain ischaemia

**Figure 2:**
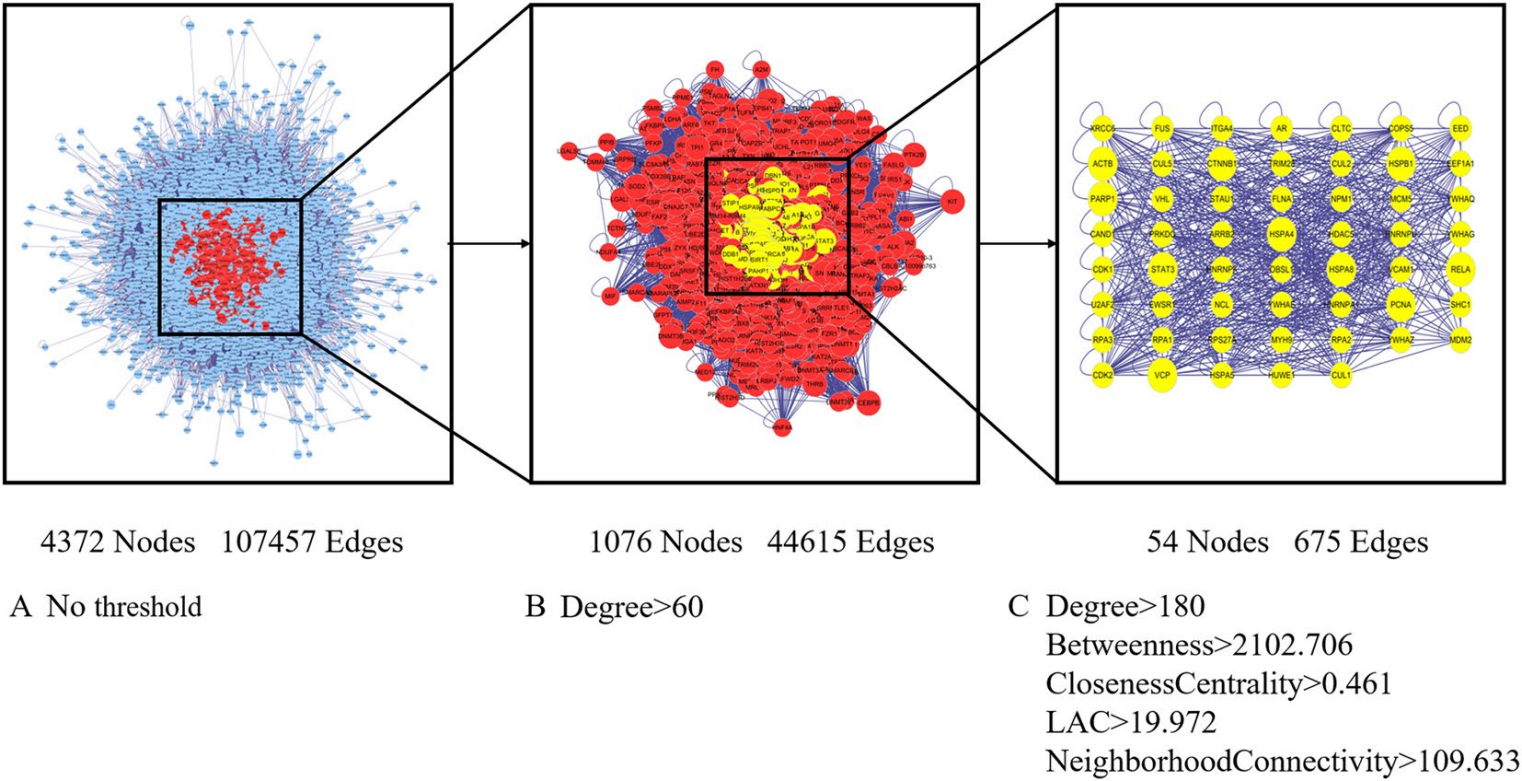
Screening process for common targets of scutellarin and brain ischaemia. The network is shown with no threshold (**A**), and Degree > 60 (**B**), Degree > 180, Betweenness > 2102.706, ClosenessCentrality > 0.461, LAC > 19.972, NeighborhoodConnectivity > 109.633 (**C**), where all edges with scores less than the threshold are removed. Visualization via Cytoscape (http://cytoscape.org/, version 3.70).

According to the PharmMapper and SwissTargetPrediction prediction results, 189 targets were predicted for scutellarin, and duplicated targets were removed. The prediction results of the OMIM, GeneCards, and DisGeNET databases were intersected to obtain a total of 721 targets for brain ischaemia. A predicted target interaction network between scutellarin and brain ischaemia was constructed using the Cytoscape, including 4372 nodes and 107,457 edges. (Fig. 2A). Nodes with a degree value greater than 2 times the median degree value were selected to build a new network, including 1076 nodes and 44,615 edges (Fig. 2B). A new network was filtered by limiting the values of Degree, Betweenness, ClosenessCentrality, LAC, and NeighborhoodConnectivity, including 54 nodes and 675 edges (Fig. 2C).

### Functional enrichment analyses

**Figure 3:**
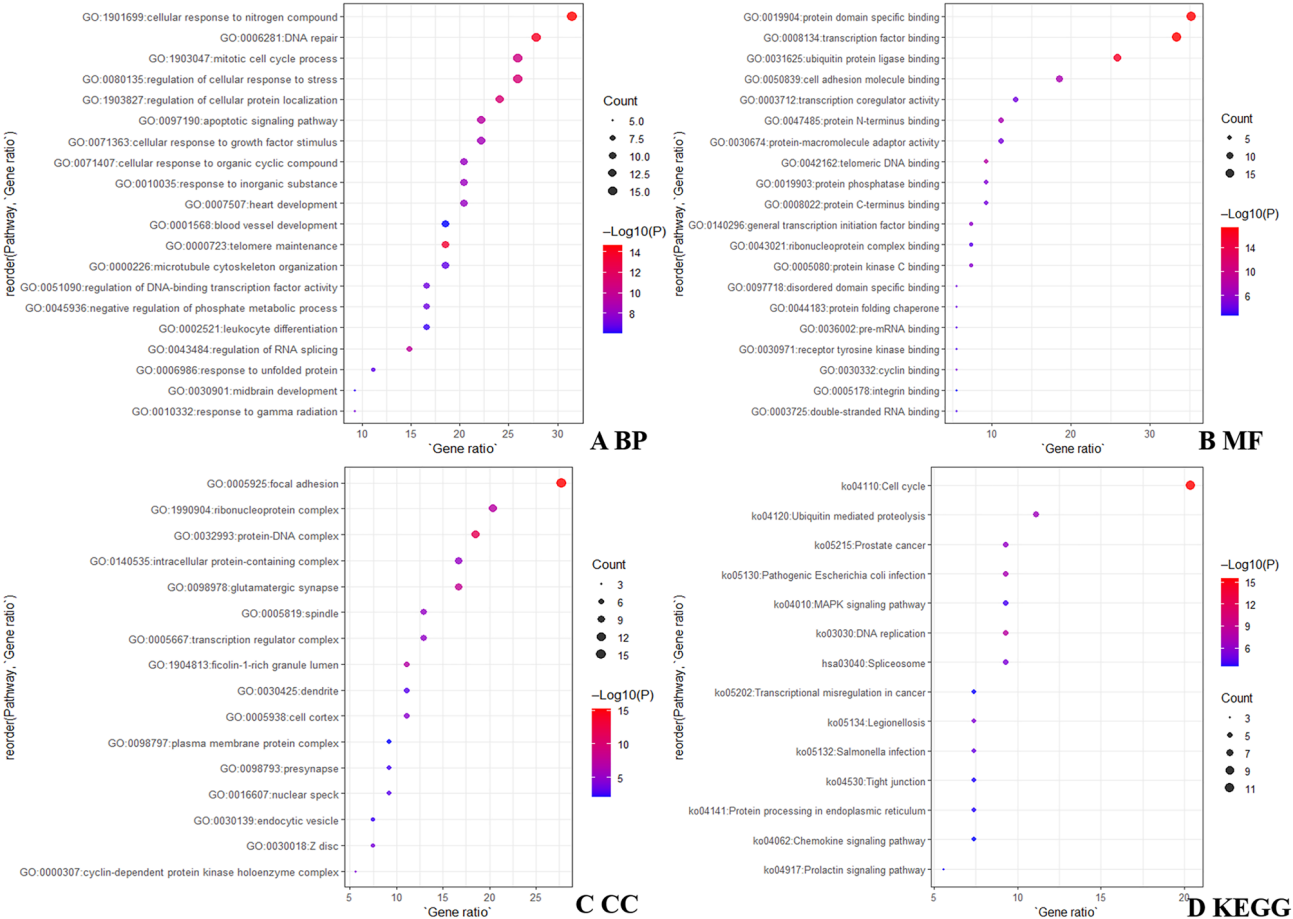
The GO and KEGG pathway enrichment bubble chart.

These 54 common targets were subjected to GO and KEGG pathway enrichment analyses using Metascape. The top 20 biological processes (BP), molecular functions (MF), and top 15 cellular components (CC) were selected by functional annotation of 54 targets, among which several BP results were mainly concentrated in cellular responses to nitrogen compounds, DNA repair, regulation of cellular response to stress, and mitotic cell cycle processes (Fig. 3A). The main molecular functions (MF) focused on protein domain-specific binding, transcription factor binding, ubiquitin protein ligase binding, and cell adhesion molecule binding (Fig. 3B). The cellular components (CC) were mainly observed in focal adhesions, ribonucleoprotein complexes, protein-DNA complexes, and glutamatergic synapses (Fig. 3C). The KEGG pathway was enriched in the cell cycle, ubiquitin-mediated proteolysis, DNA replication, and chemokine signalling pathway (Fig. 3D).

### PPI network construction and analysis

**Figure 4:**
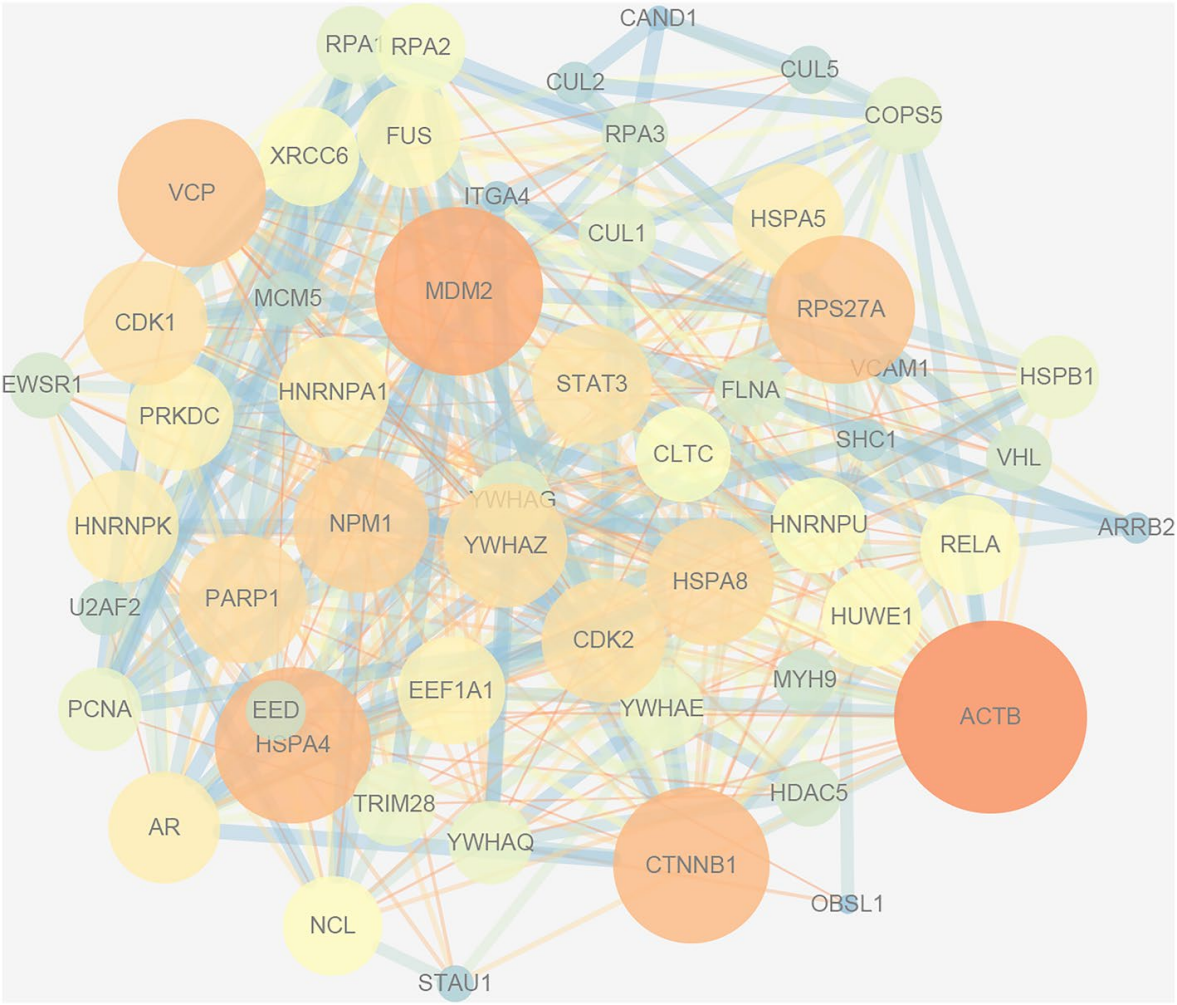
PPI network of common target proteins. Visualization via Cytoscape (http://cytoscape.org/, version 3.70).

To explore the interactions of the 54 common target proteins, a PPI network of the common target proteins was obtained from the STRING database and further visualised using Cytoscape. The resulting network incorporated 54 nodes representing 54 protein targets and 438 edges representing 438 pairs of protein interactions. Compared to the cold colour and small nodes (proteins) in the network, the warm colour and large nodes (proteins) had more edges (interactions), representing a higher degree, and are more important for further research (Fig. 4). Based on the degree values, 13 core nodes were: ACTB (degree, 40), MDM2 (degree, 34), CTNNB1(degree, 31), HSPA4(degree, 31), VCP (degree, 29), RPS27A (degree, 29), NPM1 (degree, 26), PARP1 (degree, 24), HSPA8 (degree, 24), CDK1 (degree, 23), CDK2 (degree, 23), YWHAZ (degree, 23), STAT3(degree, 22). Among them, MDM2 and RPS27A were related to ubiquitination, and CTNNB1 was associated with the regulation of cell adhesion. Notably, STAT3 was involved in the regulation of inflammatory response and is one of the key molecules involved in the biological functions of cytokines. Further screening of the KEGG pathway containing STAT3 revealed that the chemokine signalling pathway activates the JAK/STAT signalling pathway^22^, among which, the JAK2/STAT3 signalling pathway had played an important role in regulating the inflammatory response and was therefore, selected for further experimental validation.

### Target-pathway network build and analysis

**Figure 5:**
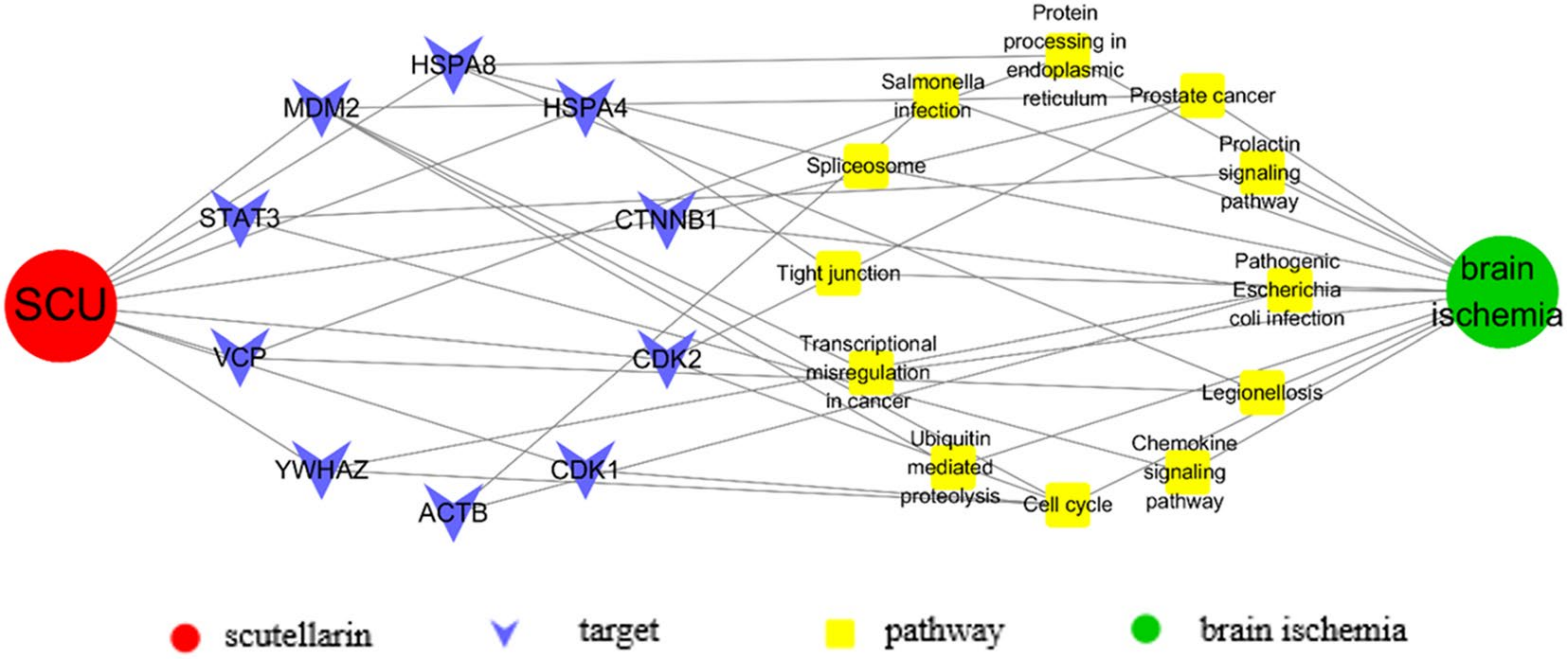
The drug–target–pathway–disease network chart.

To further reveal the relationship between scutellarin and brain ischaemia, a drug–target–pathway–disease network was constructed and visualised using Cytoscape (Fig. 5). The 13 core nodes and 14 KEGG pathways were analysed, and the final integrated network contained 10 nodes and 12 KEGG pathways, indicating that scutellarin acts on brain ischaemia through multiple targets and pathways. These results indicate that scutellarin may exert therapeutic effects via the JAK2/STAT3 signalling pathway in brain ischaemic injury. Several studies have shown that the cross-talk between microglia and astrocytes plays a pivotal role in the neuroinflammatory response. In light of this, we focused on the cerebral cortex of MCAO rats as well as BV-2 microglia-conditioned medium incubated with TNC-1 astrocytes for experimental validation both in vivo and in vitro*.*

### Effects of scutellarin on expression of JAK2/STAT3 signalling pathway proteins p-JAK2, JAK2, p-STAT3 and STAT3 in LPS-activated BV-2 microglia-mediated astrocytes

**Figure 6:**
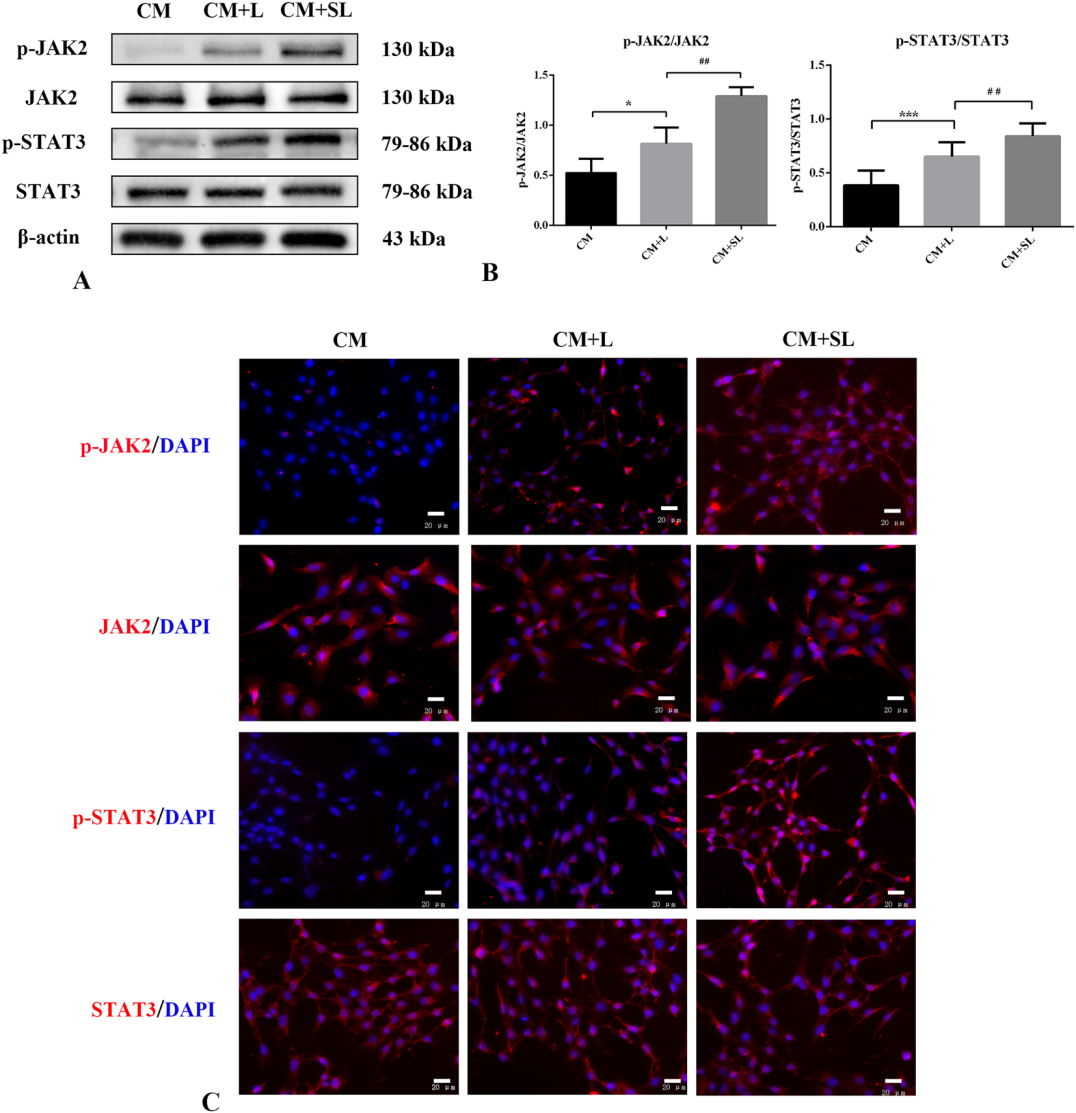
Effects of scutellarin on the expression level of JAK2/STAT3 signalling pathway proteins in TNC-1 astrocytes (n = 3). (**A**) Western Blot images (**B**) Quantitative analysis (**C**) immunofluorescence images (scale bar 20 μm) *p < 0.05 versus CM; ***p < 0.01 versus CM; ##*p* < 0.01 versus CM + L.

Astrocytes were incubated with different microglia-conditioned medium for 24 h and Western Blot results showed weak expression of p-JAK2 and p-STAT3 in TNC-1 astrocytes in the CM group, but significantly increased in the CM + L group (*p* < 0.05). Remarkably, p-JAK2 and p-STAT3 expression levels further increased in the CM + SL group (*p* < 0.05). There were no significant differences in the expression levels of JAK2 and STAT3 between the groups (*p* > 0.05) (Fig. 6A,B). Immunofluorescence staining showed that the changes in these factors were consistent with the Western Blot results (Fig. 6C). Therefore, scutellarin may exert its therapeutic effects through the JAK2/STAT3 signalling pathway.

### Effects of AG490 on expression of JAK2/STAT3 signalling pathway proteins in BV-2 microglia-mediated astrocytes

**Figure 7:**
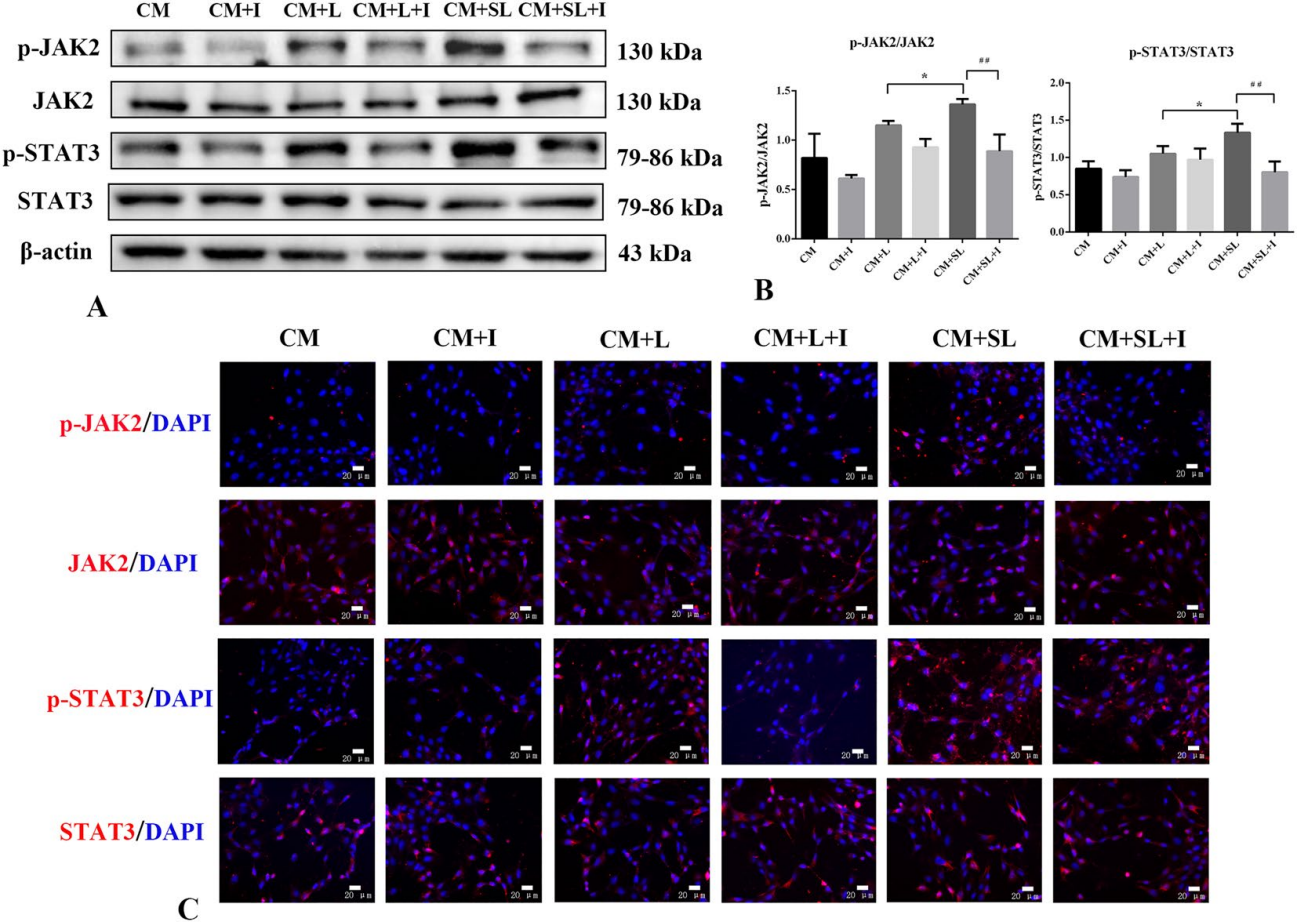
Effects of AG490 on expression of the JAK2/STAT3 signalling pathway proteins in TNC-1 astrocytes (n = 3). (**A**) Western Blot images (**B**) Quantitative analysis (**C**) immunofluorescence images (scale bar 20 μm) *p < 0.05 versus CM + L; ##*p* < 0.01 versus CM + SL.

To verify that scutellarin regulates BV-2 microglia-mediated TNC-1 astrocytes through JAK2/STAT3 signalling pathway, JAK2/STAT3 signalling pathway inhibitor AG490 (10 μM) was added to the astrocytes for 1 h, and the medium was replaced with different BV-2 microglia-conditioned medium for 24 h. Western Blotting was used to detect the expression of p-JAK2, JAK2, p-STAT3, and STAT3 in astrocytes. The expression of p-JAK2 and p-STAT3 was significantly increased in the CM + SL group (*p* < 0.05). As expected, the expression levels of p-JAK2 and p-STAT3 were significantly lower in the CM + SL + I group than in the CM + SL group (*p* < 0.01). There were no significant differences in the expression of JAK2 and STAT3 between the groups (*p* > 0.05) (Fig. 7A,B). Immunofluorescence staining showed that the changes in these factors were consistent with the Western Blot results (Fig. 7C). These results suggest that AG490 effectively inhibits the JAK2/STAT3 signalling in astrocytes, thereby neutralising the effect of scutellarin.

### Effects of scutellarin on JAK2/STAT3 signalling pathway proteins p-JAK2, JAK2, p-STAT3 and STAT3 in 1d MCAO astrocytes

Laser speckle imaging revealed a significant decrease in cerebral perfusion following MCAO (Supplementary figures: Figure S1). Western Blotting was used to detect the expression of JAK2/STAT3 signalling pathway proteins in the cerebral cortex of 1 d MCAO rats. The results showed that the expression of p-JAK2 and p-STAT3 was barely detectable in the sham-operated group; however, the expression of both biomarker proteins was significantly increased in the 1 d MCAO group compared to the sham-operated group (*p* < 0.01). In the 1 d MCAO + S group, p-JAK2 and p-STAT3 protein expression was further augmented compared with the MCAO group (*p* < 0.05). JAK2 and STAT3 expression levels were not significantly different between the groups (*p* > 0.05) (Supplementary figures: Figure S2A,B). The immunofluorescence results showed the same trend (Supplementary figures: Figure S2C,D,E,F).

### Effects of scutellarin on JAK2/STAT3 signalling pathway proteins p-JAK2, JAK2, p-STAT3 and STAT3 in cerebral cortical tissue in 3d MCAO rat

Western Blotting was used to investigate the effects of scutellarin on the JAK2/STAT3 signalling pathway proteins in the cerebral cortex of 3 d MCAO rats. The expression of p-JAK2 and p-STAT3 was hardly detected in the sham-operated group. However, the expression of both markers was significantly higher in the 3 d MCAO group than in the sham-operated group (*p* < 0.05). In the MCAO + S group, p-JAK2 and p-STAT3 expression was markedly increased, which was significantly different from that observed in the MCAO group (*p* < 0.05). There were no significant differences in the expression levels of JAK2 or STAT3 among all groups (*p* > 0.05) (Supplementary figures: Figure S3A,B). The immunofluorescence results showed the same trend (Supplementary figures: Figure S3C,D,E,F).

### Effects of scutellarin on JAK2/STAT3 signalling pathway proteins p-JAK2, JAK2, p-STAT3 and STAT3 in cerebral cortical tissue in 7d MCAO rat

**Figure 8:**
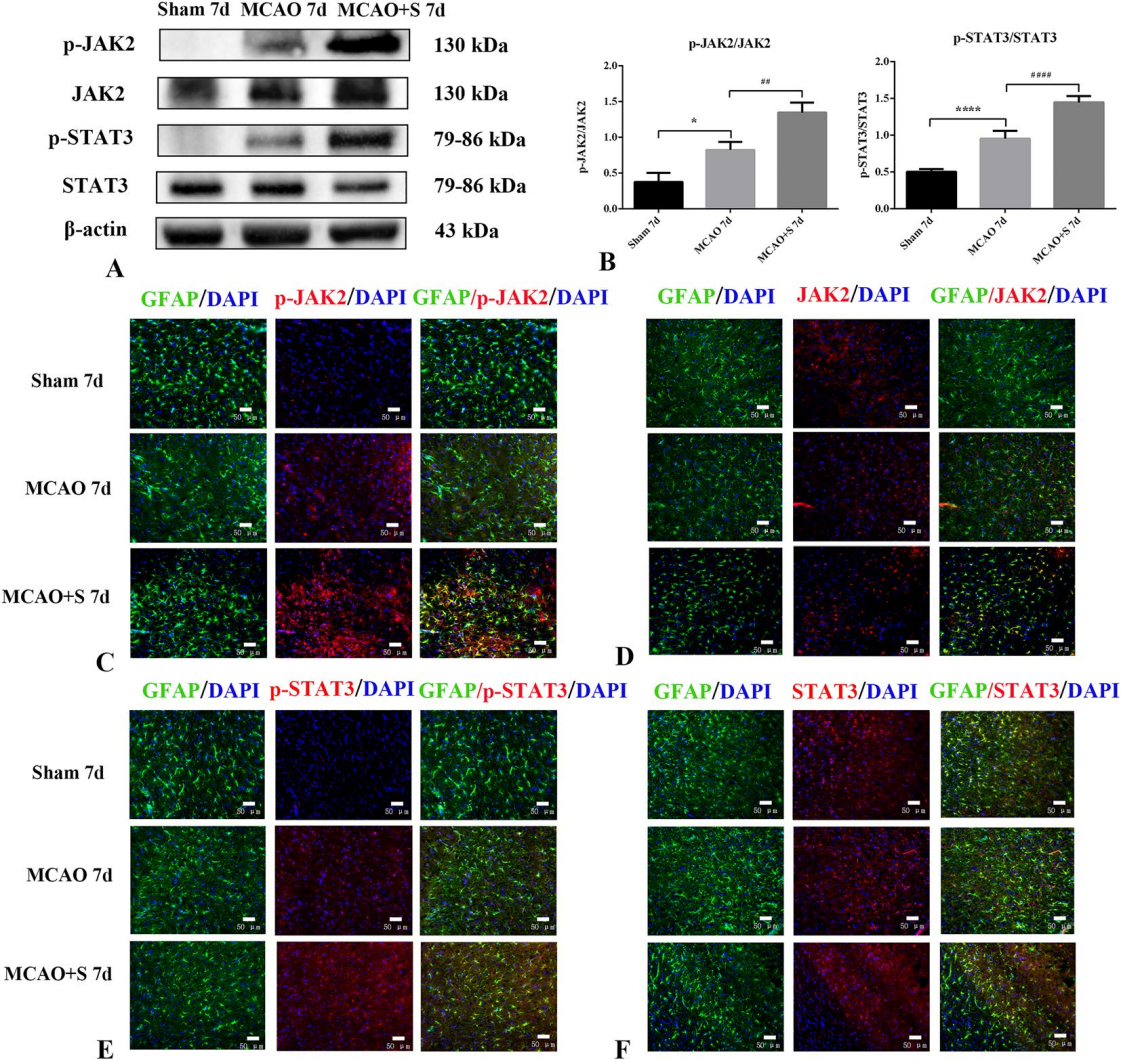
Scutellarin enhances of scutellarin on the expression of JAK2/STAT3 signalling pathway proteins in cortical tissue of 7d MCAO rats (n = 3). (**A**) Western Blot images (**B**) Quantitative analysis (**C**, **D**, **E**, **F**) immunofluorescence images (scale bar 50 μm) *p < 0.05 vs sham-operated group; ****p < 0.01 versus sham-operated group; ##*p* < 0.01 versus MCAO group; ####*p* < 0.01 versus MCAO group.

The effect of scutellarin on JAK2/STAT3 signalling pathway proteins in cerebral cortical tissue of 7 d MCAO rats was investigated by Western Blotting; p-JAK2 and p-STAT3 expression was negligible in the sham-operated group, but it was significantly increased in the 7 d MCAO group (*p* < 0.05). In rats administered scutellarin, the expression of p-JAK2 and p-STAT3 was further elevated and was significantly higher than in the MCAO group (*p* < 0.01). The expression levels of JAK2 and STAT3 were not significantly different among all groups (*p* > 0.05) (Fig. 8A,B). The immunofluorescence results were consistent with the Western Blotting results (Fig. 8C,D,E,F). Taken together, these results indicated that scutellarin consistently regulated the expression of JAK2/STAT3 signalling pathway proteins in the cerebral cortex of MCAO rats at different time points.

## Discussion

Ischaemic stroke has become a major public health concern in recent years^23^. To explore the mechanisms underlying the effects of scutellarin on cerebral ischaemia, a network pharmacology approach was used in the present study to systematically interpret the potential targets and related mechanisms of scutellarin in ischaemic stroke. Based on network analysis and literature reports, the JAK2/STAT3 signalling pathway was selected for experimental verification. In vitro experiments showed that the LPS-activated BV-2 microglia-conditioned medium activated the astrocytic JAK2/STAT3 signalling pathway. Scutellarin pretreatment further enhanced the protein expression of p-JAK2 and p-STAT3. In vivo experiments showed that the expression of p-JAK2 and p-STAT3 in rats at 1, 3, and 7 d in the MCAO group was higher than that in the sham-operated group. This increase was further enhanced by scutellarin, suggesting that the therapeutic effect of scutellarin on cerebral ischaemia is at least partially mediated by activation of the JAK2/STAT3 signalling pathway in astrocytes.

However, several studies have suggested that aberrant activation of the JAK2/STAT3 signalling pathway contributes to the inflammatory response in ischaemic stroke^24,25^ and that inhibition of the JAK2/STAT3 signalling pathway has a neuroprotective function in ischaemic stroke^26^. Nevertheless, pharmacological treatment of ischaemic stroke is a comprehensive and complex process. It has been shown that activated STAT3 leads to oxidative stress and mitochondrial dysfunction^27^. However, flavonoids also exhibit antioxidant activity. We hypothesised that scutellarin activates the JAK2/STAT3 signalling pathway, which, in turn, leads to a series of downstream cascades; however, scutellarin blocks downstream adverse factors of the JAK2/STAT3 signalling pathway, such as oxidative stress, and promotes favourable factors for the JAK2/STAT3 signalling pathway.

Curcumin, a natural polyphenol, inhibits abnormal activation of STAT3 signalling^28^. In another study, we found that scutellarin significantly reduced STAT3 expression when acting alone on astrocytes or microglia (data not yet published). In this study, LPS-activated microglia were pretreated with scutellarin and astrocytes were incubated with microglia-conditioned medium. In in vitro experiments, there was cross-talk between microglia and astrocytes, and in vivo, there was cross-talk between multiple cells types (microglia, astrocytes, and neurons), all of which may have multiple cytokine effects. We hypothesised that these factors may be responsible for the elevated STAT3 expression levels.

Despite the encouraging results of our study, there were some limitations. First, our results do not prove that scutellarin plays a neuroprotective role by activating the astrocytic JAK2/STAT3 signalling pathway. This is because we did not demonstrate a reduction in stroke volume after scutellarin treatment or a neuroprotective effect in animals treated with scutellarin in parallel with AG490. Second, our study showed that scutellarin promoted the activation of the JAK2/STAT3 signalling pathway, which has been shown to activate the JAK2/STAT3 signalling pathway and promotes neurological recovery after ischaemic stroke^29^. However, multiple studies have shown that the activation of the JAK2/STAT3 signalling pathway plays a key role in ischaemic brain injury^30–32^. Clearly, our study supports the former view; however, further research is needed to interpret this. Third, it has been shown that oxidative stress, inflammation, and apoptosis interact with each other and form a complex signalling network that leads to neuronal cell necrosis, senescence and apoptosis^33–35^. However, previous studies have shown that scutellarin has antioxidant effects. Specific experimental validation has not been performed, and the downstream mechanisms of scutellarin after the completion of antioxidation are not known. However, further studies are required to confirm this hypothesis.

In conclusion, this study systematically analysed the possible targets and mechanisms of action of scutellarin in cerebral ischaemia. We experimentally verified the therapeutic effect of scutellarin on cerebral ischaemia by activating the astrocytic JAK2/STAT3 signalling pathway. We are confident that our study provides a reliable scientific basis for the clinical application of scutellarin in the prevention and treatment of brain ischaemia, and perhaps also in other ischaemic cardiovascular diseases. However, downstream mechanisms still need to be explored.

## Methods

### Bioinformatics analyses

#### Target prediction

To identify the corresponding targets of scutellarin, the PharmMapper (http://www.lilab-ecust.cn/pharmmapper/)^36^ and SwissTargetPrediction (http://www.swisstargetprediction.ch/)^37^ databases were searched to predict related protein targets. These protein names were then entered into the UniProt database (http://www.uniprot.org/)^38^ to obtain their official gene names. A hundred scutellarin targets were obtained from SwissTargetPrediction. In total, 109 scutellarin targets were selected from PharmMapper (z-score > 0.5). Finally, 189 scutellarin targets were identified by combining the two databases. Disease targets for brain ischaemia were obtained from three databases: OMIM (https://omim.org/)^39^, GeneCards (https://www.genecards.org/)^40^ and DisGeNET (http://www.disgenet.org)^41^. In total, 401 brain ischaemia targets were obtained from the OMIM database, 200 from the GeneCards database (Relevance score > 13.34), and 202 from the DisGeNET database (Score > 0.02). Finally, a total of 721 targets for brain ischaemia were obtained by taking the ensemble from the three databases.

#### Network construction

An intersection network of scutellarin and brain ischaemia was constructed using Cytoscape (http://cytoscape.org/, version 3.70)^42^. First, brain ischaemia and scutellarin networks were constructed based on the obtained targets. Second, the intersection of the two networks was used to construct a merged network, and the values of degree, Betweenness, ClosenessCentrality, LAC, and NeighborhoodConnectivity were limited to filter a new network. The merged network of scutellarin and brain ischaemia comprised 54 nodes and 675 edges.

#### Protein–protein interaction data

Protein–protein interaction (PPI) data were extracted from the STRING database (https://string-db.org/)^43^, which is a database of known and predicted protein–protein interactions that stem from computational prediction, including both direct and indirect interactions among proteins. Ultimately, the PPI data with species limited to “Homo sapiens” and confidence scores of 0.9 were reserved for further research. Visualization via Cytoscape 3.70.

#### Enrichment analysis

The Metascape (http://metascape.org)^44^ online database was used to perform functional enrichment analyses, including Gene Ontology (GO) and Kyoto Encyclopedia of Genes and Genomes (KEGG) pathway enrichment analyses. GO analysis included biological processes (BP), cellular components (CC), and molecular functions (MF). Statistical significance was set at *p* < 0.01.

#### Construction of drug-target-pathway-disease network chart

Ten core targets and 12 related pathways were selected, and the results were imported into Cytoscape to construct a drug-target-pathway-disease network.

### Ethics statement

All animal experiments were performed in accordance with the ARRIVE guidelines (https://arriveguidelines.org/arrive-guidelines) and the Guide for the Care and Use of Laboratory Animals at Kunming Medical University. All experimental protocols and animal use were approved by the Laboratory Animal Ethics Committee of Kunming Medical University (approval no. kmmu20220066). Efforts were made to minimise the number of rats used and their suffering.

### Animals and experimental groups

Adult male Sprague–Dawley rats (*SD*, 250–280 g, 8 weeks old) were group-housed with a 12-h light‑dark cycle and provided chow and water ad libitum. They were randomly divided into sham-operated + saline (sham), middle cerebral artery occlusion (MCAO) + saline (MCAO), and MCAO + scutellarin (MCAO + S) groups. In the MCAO group, anaesthesia was induced by an intraperitoneal injection of sodium pentobarbital (50 mg/kg). A dental drill was used to create a circular aperture in the right parietal bone to expose the underlying main trunk of the MCA. The right MCA was occluded by electrocoagulation. The rats in the MCAO + S groups were administered an intraperitoneal injection of scutellarin (100 mg/kg, as described in our previous study^15^) dissolved in saline 2 h before and 12, 24, 48, and 60 h after MCAO; the other two groups were injected with saline. The rats were sacrificed 1, 3, and 7 d after MCAO. Laser speckle imaging system (RFLSI III; RWD) to measure cerebral blood perfusion.

### Cell culture and preparation of conditioned medium

Both TNC-1 astrocytes and BV-2 microglia were purchased from the American Type Culture Collection (ATCC, USA). TNC-1 astrocytes and BV-2 microglia were cultured in Dulbecco’s Modified Eagle Medium (DMEM) supplemented with 10% foetal bovine serum (FBS) at 37 °C in a humidified incubator under 5% CO_2_. Upon reaching confluence, the BV-2 microglial culture medium was changed to DMEM without FBS. Three different BV-2 conditioned medium were extracted: CM, BV-2 cells were cultured in DMEM for 3 h; CM + L, BV-2 cells treated with 1 µg/mL of LPS in DMEM for 3 h; CM + SL, BV-2 cells were treated with 0.54 mmol/L of scutellarin for 1 h. The drug dose was selected based on our previous results^15^. After scutellarin, BV-2 cells were treated with 1 µg/mL of LPS in DMEM for 3 h. All conditioned medium were collected and transformed into 6-well plates or 24-well plates with TNC-1 astrocytes grown to confluence for 24 h. Astrocytes were then divided into different groups depending on the microglia-conditioned medium added. When astrocytes reached the degree of convergence, some were treated with the JAK2/STAT3 signalling inhibitor AG490 for 1 h before the addition of microglia-conditioned medium. AG490 is a specific inhibitor of the JAK2/STAT3 signalling pathway that can effectively inhibit JAK2 phosphorylation, and subsequently inhibit STAT3 phosphorylation. Based on this, astrocytes were divided into six groups comprising CM, AG490 group (CM + I), CM + L, CM + L + I, CM + SL and CM + SL + I group by incubating them with the above conditioned medium for 24 h. After various treatments, astrocytes were collected for protein extraction or immunofluorescence analysis.

### Western blot

Rat cerebral cortices in each group were lysed on ice with precooled lysis buffer (Dalian Meilunbio) to prepare tissue homogenates, and cultured TNC-1 astrocytes were lysed similarly. Cells were harvested 15 min after cleavage using a cell scraper. The tissue homogenate and cell protein were centrifuged at 14,000 rpm at 4 °C for 20 min, and the supernatant was collected. The protein concentration was determined using a BCA protein assay kit (Dalian Meilunbio). Protein samples were loaded and heated at 95 °C for 10 min and separated by SDS-PAGE electrophoresis. Protein bands were electro-blotted onto polyvinylidene difluoride (PVDF) membrane and blocked with 5% skim milk powder for 2 h. Membranes were cut prior to hybridisation with antibodies: p-JAK2 (1:1000), JAK2 (1:1000), p-STAT3 (1:2000), STAT3 (1:1000) (cat.nos.3776, 3230, 9145, and 12,640 respectively; Cell Signaling Technology) and β-actin (1:5000; cat.no.66009–1-Ig; Protein-tech). After 16 h of incubation with the respective primary antibodies, the membrane was incubated with the following secondary antibodies: goat anti-rabbit IgG-HRP and goat anti-mouse IgG-HRP secondary antibody (1:5000; cat. nos. SA00001-2, SA00001-1 respectively; Protein-tech). Specific binding was revealed using an ECL chemiluminescence kit (Biosharp Life Sciences) and a gel imaging system (Bio-Rad). All experiments were repeated three times.

### Immunofluorescence staining

Rats were deeply anaesthetised with 3% sodium pentobarbital and then sacrificed by perfusion with 4% paraformaldehyde in 0.1 M phosphate buffer. The brains were removed and embedded in an OCT-embedding agent. Coronal sections of the brain were cut at 10 μm thickness on a microtome. The sections were then rinsed with PBS. To block non-specific binding proteins, tissue sections were incubated in 5% goat serum diluted in PBS for 1 h at room temperature. Tissue sections were incubated with the following primary antibodies: GFAP (1:200; cat.no. mab360; Sigma), p-JAK2 (1:1000; cat.no. ab32101; Abcam), JAK2 (1:150; cat.no. ab108596; Abcam), p-STAT3 (1:100) and STAT3 (1:1000) (cat. nos. 9145 and 12,640 respectively; Cell Signaling Technology). After 16 h, the brain sections were washed with PBS and incubated with fluorescent secondary antibodies: Cy3-conjugated secondary antibody (1:200) and CoraLite488-conjugated secondary antibody (1:200) (cat. nos. SA00009-2 and SA00013-1 respectively; Protein-tech) for 1 h at room temperature. After washing with PBS, fluorescent sealing tablets containing DAPI were used to seal the sections. Images were captured using a Zeiss microscope (Axio observer Z1). The flowchart of the experiment is illustrated in Fig. 1.

### Statistical analyses

Data are presented as mean ± standard deviation (m ± SD). After normality and homoscedasticity of variance tests, one-way ANOVA and Tukey post hoc test were used. Statistical significance was set at *p* < 0.05. GraphPad Prism 6 software was used to analyse the data.

## Supplementary Information

Supplementary Information.

## Data Availability

The datasets used and/or analyzed during the current study are available from the corresponding author on reasonable request.
