# Supplementary material for: Network pharmacology to explore the mechanism of scutellarin in the treatment of brain ischaemia and experimental verification of JAK2/STAT3 signalling pathway
Source: Sci Rep. 2023 May 9;13:7557. doi: 10.1038/s41598-023-33156-5 (PMC10169761; doi:10.1038/s41598-023-33156-5)
Supplement: Supplementary file 1 — Supplementary Information. [file 41598_2023_33156_MOESM1_ESM.docx]

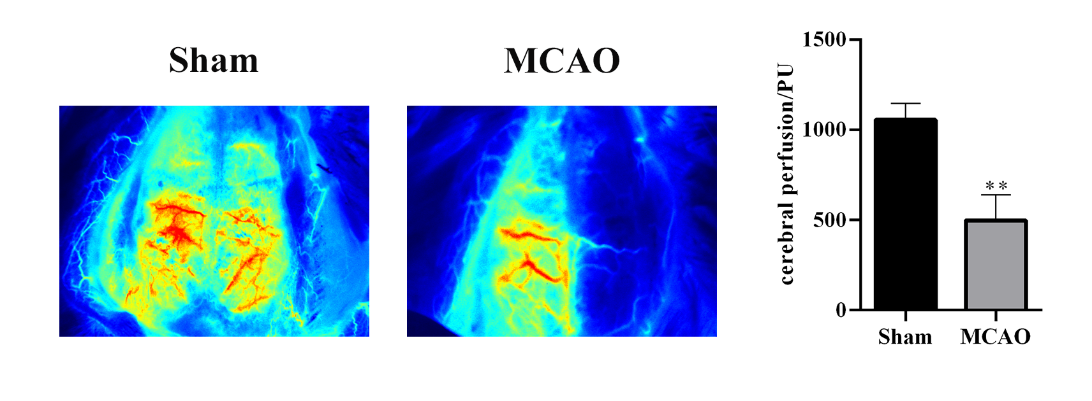


Fig.S1 Laser Speckle imaging (n=3). ***p*<0.01 vs sham group. Visualization via RFLSI Analysis (<https://www.rwdls.com/>, version 2.0.25.25018).


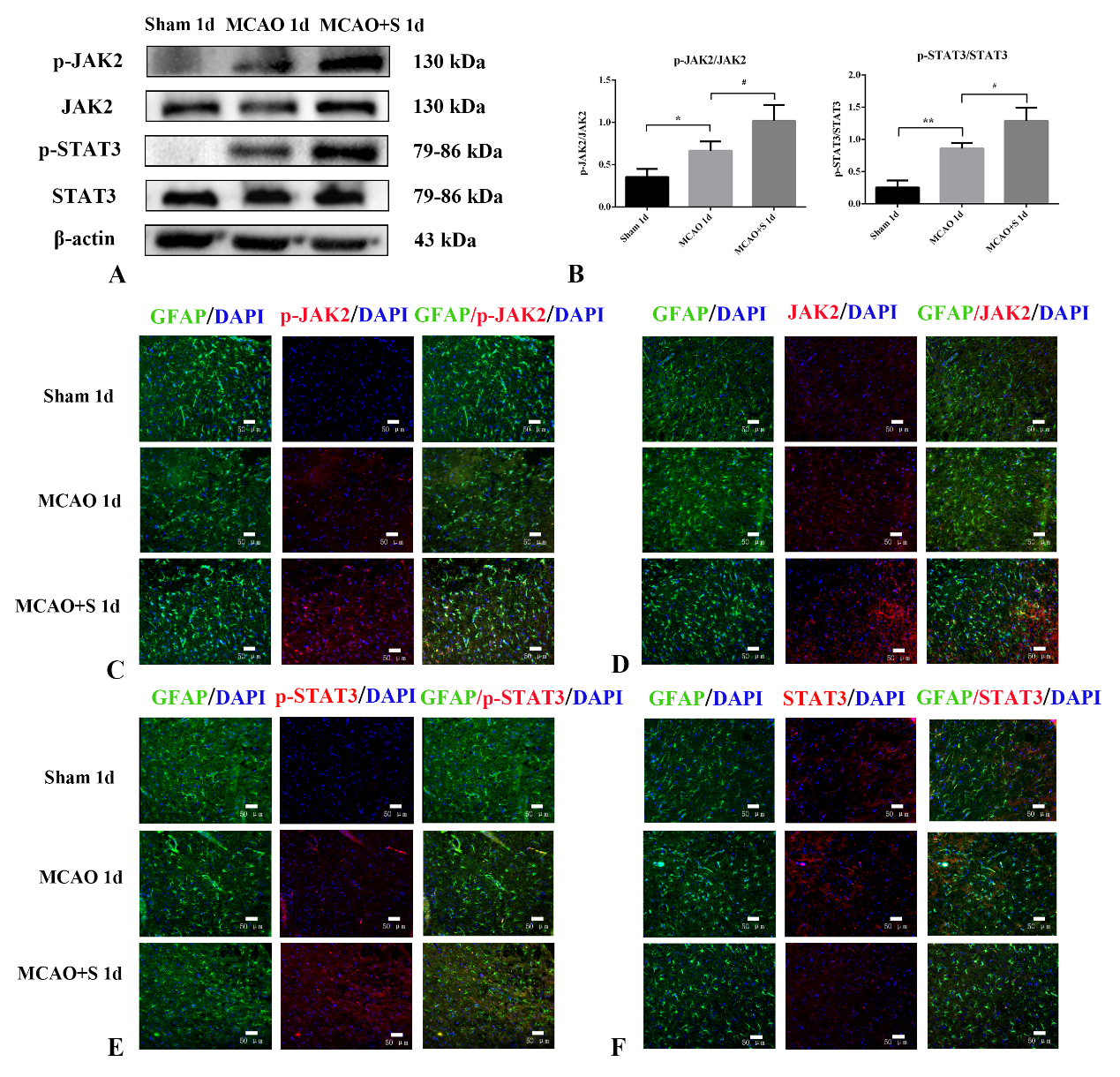


Fig.S2 Scutellarin enhances of scutellarin on the expression of JAK2/STAT3 signalling pathway proteins in cortical tissue of 1 d MCAO rats (n=3).

A: Western Blot images B: Quantitative analysis C, D, E, F: immunofluorescence images (scale bar 50 μm) **p*<0.05 vs sham-operated group; ***p*<0.01 vs sham-operated group; #*p*<0.05 vs MCAO group.


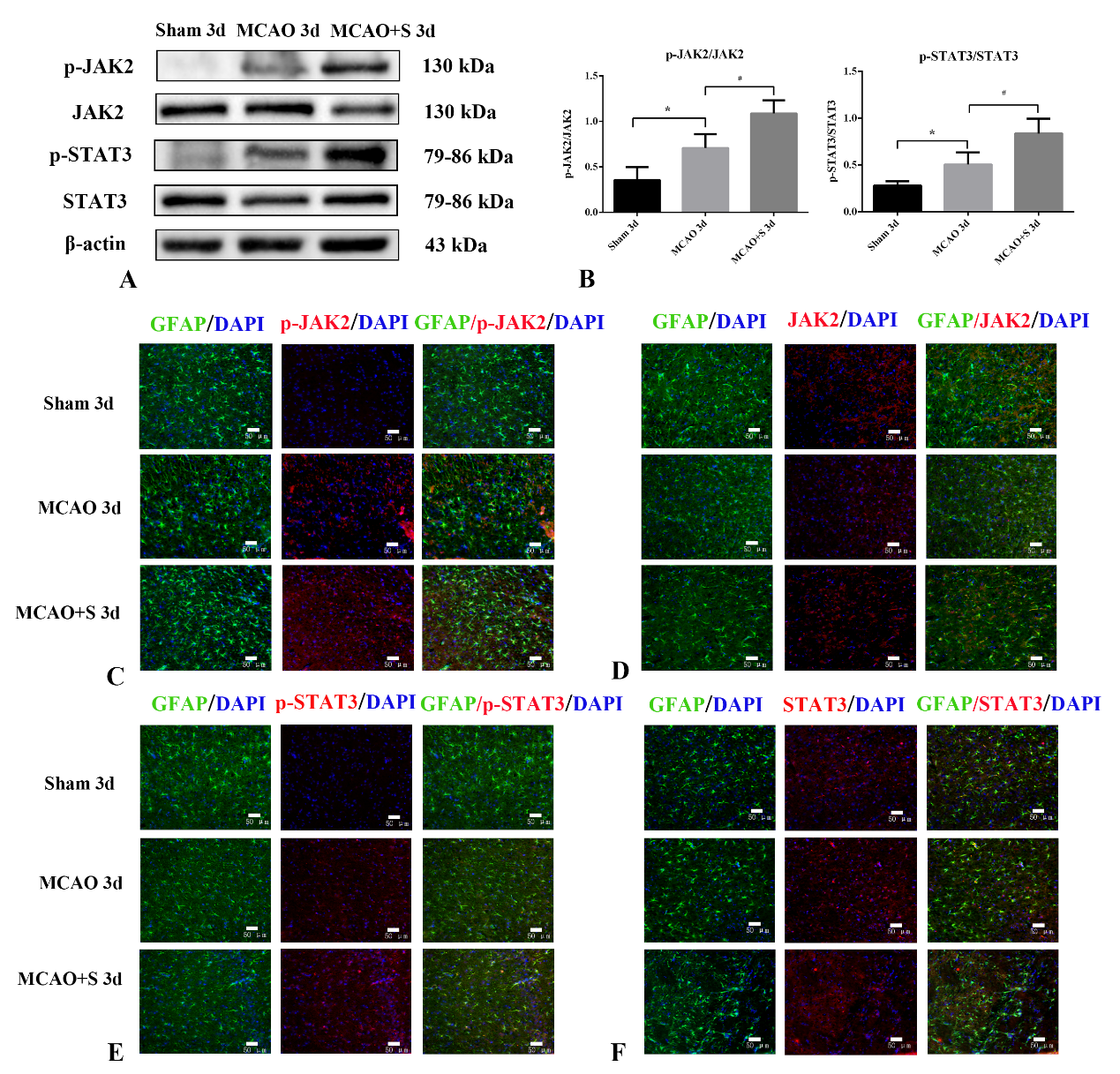


Fig.S3 Scutellarin enhances of scutellarin on the expression of JAK2/STAT3 signalling pathway proteins in cortical tissue of 3 d MCAO rats (n=3).

A: Western Blot images B: Quantitative analysis C, D, E, F: immunofluorescence images (scale bar 50 μm) **p*<0.05 vs sham-operated group; #*p*<0.05 vs MCAO group.


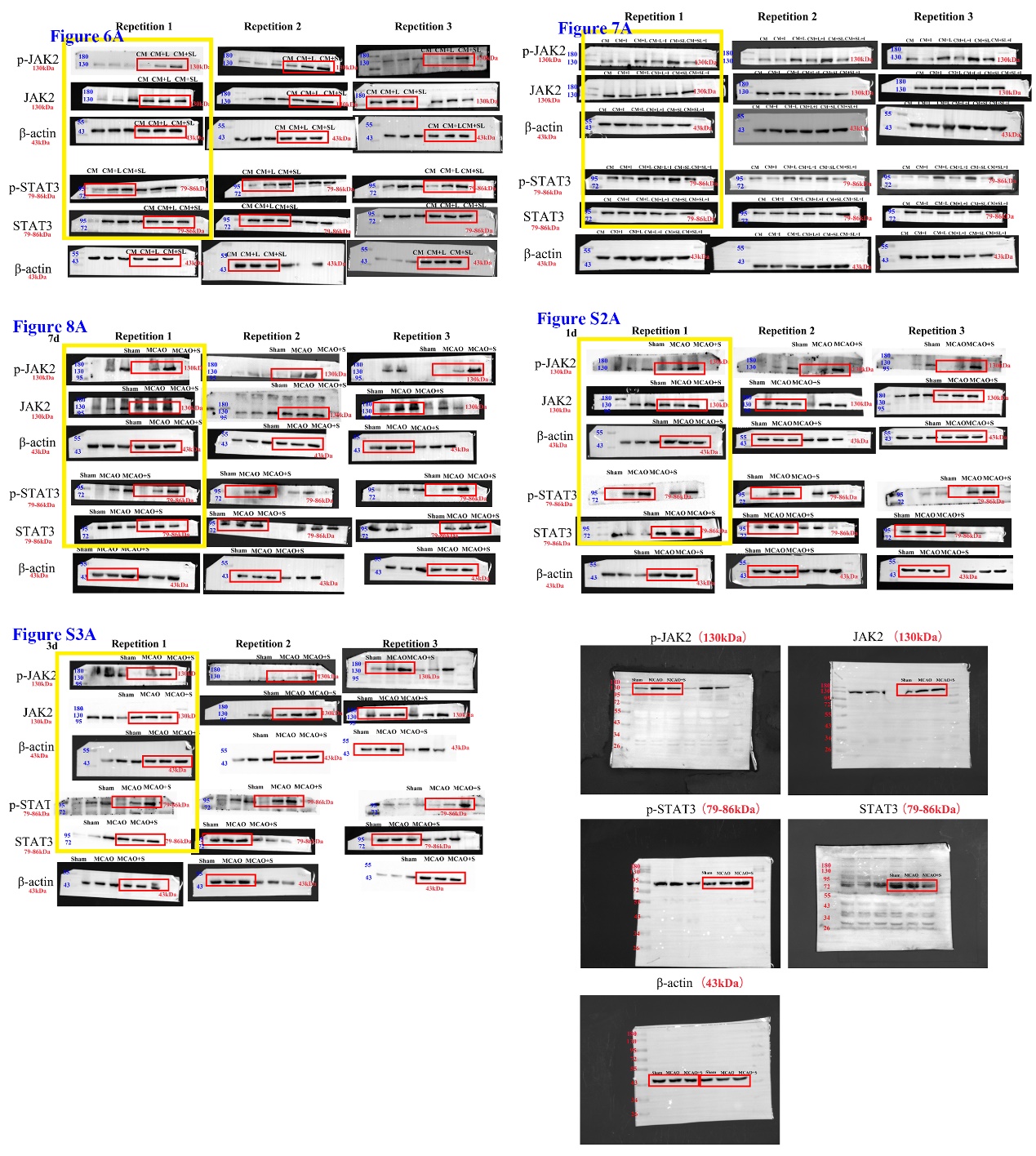


Fig.S4 Western Blot original images.
